# The Comparative Evaluation of Postoperative Pain After the Use of WaveOne Gold and TruNatomy Filing Systems in a Tooth With Irreversible Pulpitis: An Observational Study

**DOI:** 10.7759/cureus.30707

**Published:** 2022-10-26

**Authors:** Priyanka R Bhojwani, Meghna J Paryani, Nikhil Mankar, Amit Reche, Priyanka Paul, Pradnya P Nikhade

**Affiliations:** 1 Department of Conservative Dentistry and Endodontics, Sharad Pawar Dental College and Hospital, Datta Meghe Institute of Medical Sciences, Wardha, IND; 2 Department of Public Health Dentistry, Sharad Pawar Dental College and Hospital, Datta Meghe Institute of Medical Sciences, Wardha, IND; 3 Department of Dentistry, Sharad Pawar Dental College and Hospital, Datta Meghe Institute of Medical Sciences, Wardha, IND

**Keywords:** instrumentation, postendodontic pain, intensity, incidence, reciproc

## Abstract

Introduction

The present study aimed to clinically compare the incidence of postoperative pain after the endodontic treatment of posterior teeth using the WaveOne Gold (WOG) and TruNatomy (Dentsply Maillefer, Ballaigues, Switzerland) filing systems. The study gives a better understanding of the association of postoperative pain and filing systems used. The patients were selected in such a manner that they have a similar diagnosis before the initiation of treatment. It also helps to understand if the postoperative pain is dependent or independent of the filing system used.

Methods

In the study, 32 vital teeth with irreversible pulpitis with no periapical lesion were selected for the study. The patients were divided into two groups (n=16) according to the instrumentation system used (WaveOne Gold group and TruNatomy group). The treatments were performed in a single session. The participants were asked to rate the intensity of postoperative pain on a visual analog scale (VAS) (no pain, mild pain, moderate pain, and severe pain) after 24 hours, 72 hours, and seven days; the tests of significance used are Friedman test or Mann-Whitney test.

Results

The incidence of postoperative pain is comparatively less in WaveOne Gold group after 24 and 72 hours compared with those in the TruNatomy group. No postoperative pain is experienced after seven days by the participants of both groups.

Conclusions

Postoperative pain is expected more after the preparation of the root canal system with the TruNatomy as compared to WaveOne Gold.

## Introduction

Postoperative pain after root canal treatment is defined as the sensation of discomfort after endodontic intervention. A tooth may require root canal therapy for a variety of reasons. When a tooth becomes inflamed or infected to the point that it can no longer heal, root canal therapy is required. The most prevalent reason for postoperative discomfort includes mechanical preparation and obturation beyond the apex, germs not destroyed during first disinfection, and the extrusion of irrigants and debris beyond the apex [1]. The instrumentation procedure has been cited as a key contributing factor to postendodontic pain [2], which is multifactorial [3]. Debris and bacterial ejection during chemomechanical preparation may be the cause of this [4,5], which enhances the inflammatory response [6]. Irrigants and drugs must be able to reach the apical region of the canal, which requires cleaning and shaping [7]. In typical therapeutic irrigation, a conventional needle and some frequency of sonic stimulation are used [8]. Endodontic failure is caused by a poor coronal seal and the persistence of germs after root canal therapy. The amount of damage done to periradicular tissues during endodontic therapy determines the severity of discomfort. Dentin shavings, germs, irrigating solution, and the presence of pulp tissue after treatment are related to discomfort [1]. The previous studies have related postoperative pain to either instrumentation or debris extrusion, whereas the results show the slightest possibility that it could be related to other factors. Consequent edema and increased vascular permeability induce inflammatory mediators to be released in the periradicular region, resulting in nerve fiber compression and discomfort [7].

There is a relationship between debris extrusion and the presence of inflammatory mediators in clinical practice, which is regulated by root canal system preparation [9]. Extruded debris amounts vary depending on the instrumentation method utilized, but no technology has yet been able to prevent it from occurring. The goal of this study is to compare the two file systems and see if instrumentation reduces the incidence and severity of postoperative pain. WaveOne Gold (WOG) (Dentsply Maillefer, Ballaigues, Switzerland) is a file system that comes in sizes or tapers of 20/0.07, 25/0.07, 35/0.06, and 45/0.05 [10]. The design of files is two cutting edges, restricting file-dentin engagement to only 1-2 contact points [10]. The file's nickel-titanium (NiTi) alloy has been heat-treated, making it extremely flexible and fatigue-resistant [11]. WaveOne Gold (Dentsply Maillefer, Ballaigues, Switzerland) files are WaveOne-modified reciprocating single-file systems. Gold heat treatment, a novel parallelogram cross-sectional design with 85° cutting edges in contact with the canal, and a variable, diminishing taper are all used to improve WaveOne Gold system. It rotates 360° in three cycles at a speed of 350 rpm (150° counterclockwise {CCW} and 30° counterclockwise and alternating clockwise movements) [10,11-13]. Short-term pain (24-72 hours) is to be expected but will lessen after seven days, according to these findings [14]. The WaveOne Gold system uses patented heat treatment to improve the instrument's flexibility, cutting efficiency, and cyclic fatigue resistance [3,14]. These properties favor the maintenance of the path after curved root canal preparation, which justifies its use in our study.

TruNatomy (Dentsply Maillefer, Ballaigues, Switzerland) filing system is now available as orifice modifier (20/0.08 purple), glider (17/0.02 white), prime shaping file (26/0.04 taper, red), medium shaping file (36/0.03 taper, green), and small shaping file (20/0.04 taper, yellow) [15]. The TruNatomy device is employed in continuous rotation movement, as suggested by the manufacturer. Increased rotation can produce debris extrusion and periodontal tissue injury. The instrument is made of annealed heat-treated fire wire, a nickel-titanium alloy. According to the manufacturer, TruNatomy files provide slender shaping and improved debridement because of the extra space they produce [16].

Through the instrument's geometry, regressive tapers, slender design, and heat treatment of the alloy, the TruNatomy system preserves the tooth's integrity with the maximum preservation of paracervical dentin [11,15-17]. The file's parabolic cross section combines great efficiency and flexibility with safety and resistance to fracture in curved canals. There is a need for a study that not just confirms the relation by the association of the postoperative pain and filing system but also compares the two filing systems. This study not only helps to overcome this research gap but also gives a better understanding of postendodontic pain in relation to the filing system. The objective is to examine the occurrence of postoperative pain of the mandibular posterior teeth using WaveOne Gold and TruNatomy filing systems after endodontic treatment in a clinical setting.

## Materials and methods

Study setting and design, participant, and instrument

The study is conducted in Sharad Pawar Dental College (SPDC), in the Department of Conservative Dentistry and Endodontics, for a period of six months. A total of 32 patients in the age group of 20-60 years old were selected for the study. The patients are allocated into two groups (Table 1).

**Table 1:** Allocation of the participants.

Group	Allocation
Group 1	Instrumentation by using WaveOne Gold file system (Dentsply Maillefer, Ballaigues, Switzerland)
Group 2	Instrumentation by using the TruNatomy filing system (Dentsply Maillefer, Ballaigues, Switzerland)

The study involves patients who desire single-visit root canal treatment, tooth present without periapical lesions, teeth with root fillings that show radiographic indications of periradicular illness, and teeth with sufficient crown structure for rubber dam isolation; only mandibular molars are used in this study. Multiple-seating root canal treatment, the presence of a periodontal pocket greater than 4 mm deep, patients who are medically compromised or have medicine allergies, patients with any systemic diseases, tooth present with periapical lesions, and root fracture tooth are excluded to avoid bias.

Sample size calculation

On the basis of the comparative study by Xavier et al. (Postoperative pain after use of the WaveOne Gold and XP-endo Shaper systems: a randomized clinical trial) [14], the mean and standard deviation for postoperative pain in the WaveOne Gold group at 24 hours were 0.68 and 1.55, respectively, and the mean and standard deviation for postoperative pain in WaveOne Gold group at seven days were 0.00 and 0.00, respectively; therefore, the mean difference between 24 hours and seven days is 0.68. The pooled standard deviation between 24 hours and seven days was 1.55/2=0.775.

Data collection

Each of the 32 patients was taught how to use a visual analog scale (VAS), which is a psychometric response scale that can be used for the subjective evaluation of pain and is used in this study to determine their preoperative and postoperative pain levels before starting therapy. VAS is a horizontal line with values divided into visual categories that have been tested previously. On a scale of 1 to 10, patients are asked to rate their pain: no pain (0), mild pain (1-3), moderate pain (4-6), and severe pain (7-10) [18]. The postoperative pain is evaluated at three intervals, 24 hours, 72 hours, and seven days.

Ethical consideration

The research ethics committee gave its clearance to this observational study (reference number: DMIMS(DU)/IEC/2022/984). To take part in the trial, every patient signed an informed consent form. During treatment, all specialists adhered to this agreed regimen.

Procedure

Local anesthesia is given with 2% lignocaine and epinephrine (1:100,000). Access cavities are prepared using small round bur (Mani BR-41) on a high-speed endodontic motor. Once access has been gained, both groups build a glide path up to K-file number 15, using push-and-pull motion. The working length is measured with Root ZX II (J. Morita USA, Inc., Irvine, CA) apex locator up to the apical foramen, at which point the file is removed and the length is measured with an endodontic ruler. The working length was verified by radiographic confirmation. Each group follows the manufacturer's recommended instrumentation order. All instruments are driven by a torque-restricted endo motor.

Group 1

WaveOne Gold group: In reciprocating mode, WaveOne Gold (Dentsply Maillefer) file (main file: size 25 and 0.07 tapers) is pushed into root canal until resistance is encountered. Three back-and-forth movements are conducted after that, with slight apical pressure applied. These steps are carried out again and again until working length is obtained.

WaveOne Gold's instrumentation (WaveOne Gold filing system): We'll employ the WaveOne Gold glider file (15/0.02 variable taper). The X-Smart Plus (Dentsply Maillefer, Ballaigues, Switzerland) endodontic motor is set for reciprocating action (150° counterclockwise and 30° clockwise) as the WaveOne Gold main tool (25/0.07) is used to prepare the canals. As accordance with the manufacturer's recommendations, filing is slowly performed in an in-and-out pecking motion. Up until the working length is attained, this protocol is repeated. After third peck, the file is removed from the canal, the flutes of the file are cleaned, and canal is irrigated.

Group 2

TruNatomy group: Instead of 1.2 mm NiTi wire, the TruNatomy (Dentsply Maillefer, Ballaigues, Switzerland) file system uses a special 0.8 mm NiTi heat-treated wire. The file has design that provides increased flexibility and cyclic fatigue resistance, as well as a thin design and superior canal-centering capabilities, and has a regressive taper.

Endodontic motor (X-Smart Plus), set at 500 rpm/1.5 Ncm according to the manufacturer's instructions, is used to run the rotary instrumentation with TruNatomy files. A TruNatomy orifice modifier (20/0.08) is used in the coronal third, followed by a TruNatomy glider (17/0.02) and the principal instrument (26/0.04). The file is removed, its flutes are cleaned, and the canal is irrigated after two to three gentle 2-5 mm motions are used to introduce all of the files. The process is repeated until it reaches the required length. The primary instrument and the glider use whole working length. Each root canal received a final flush of 5 ml sterile distilled water using a plastic syringe and a 30-gauge needle tip that was 2 mm too short for the operational length. With AH Plus (Dentsply Maillefer, Ballaigues, Switzerland) epoxy resin sealer, root filling is performed using a single-cone technique, and the gutta-percha cone is suited to the instrument used in each group (Dentsply Maillefer, Ballaigues, Switzerland). Periapical radiography is used to assess the effectiveness of root filling. All patients' occlusions are assessed, and if necessary, high spots are rectified using carbon paper. Post therapy, 600 mg ibuprofen was given as and when needed. The incidence of postoperative pain as assessed by VAS was compared between two groups, and three time points are studied, i.e., 24 hours, 72 hours, and seven days.

Statistical analysis

All the results are calculated by using Statistical Package for the Social Sciences (SPSS) version 24 (IBM SPSS Statistics, Armonk, NY). Descriptive statistics was performed for mean and standard deviation. Inferential statistics was calculated for finding the significance between two groups compared and tested at p=0.05. Intergroup comparison is done by Mann-Whitney test, and Friedman test is used for determining the mean at various time intervals. The null hypothesis here is a result that shows no difference in postoperative pain in relation to both groups.

## Results

**Figure 1:**
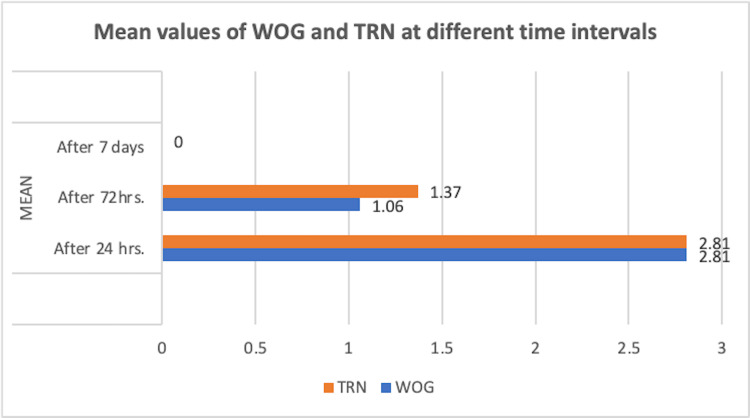
Mean values of WOG and TRN at different time intervals. TRN: TruNatomy; WOG: WaveOne Gold

According to the parameters set forth in this study, despite the fact that TruNatomy suffers from more postoperative pain at 24 hours, 72 hours, and seven days, there was a statistically insignificant difference in postoperative discomfort between the preparation systems studied (Table 2 and Figure 1).

**Table 2:** Descriptive statistics for WaveOne Gold and TruNatomy. S: significant

Treatment		N	Minimum	Maximum	Mean	Standard deviation	Friedman test (chi-square value)	P-value
WaveOne Gold	After 24 hours	16	2.00	5.00	2.81	0.83	29.7	<0.01*S
	After 72 hours	16	0.00	2.00	1.06	0.77		
	After seven days	16	0.00	0.00	0.00	0.00		
	Valid N (listwise)	16						
TruNatomy	After 24 hours	16	2.00	5.00	2.81	1.10	29.5	<0.01*S
	After 72 hours	16	0.00	2.00	1.37	0.71		
	After seven days	16	0.00	0.00	0.00	0.00		
	Valid N (listwise)	16						

Mean ranks at various time intervals

Table 3 shows the mean ranks at various time intervals.

**Table 3:** Mean ranks at various time intervals. TRN: TruNatomy; NS: Nonsignificant

	Treatment	N	Mean rank	P-value
After 24 hours	WaveOne Gold	16	17.19	0.65*NS
	TRN	16	15.81	
	Total	32		
After 72 hours	WaveOne Gold	16	14.69	0.23*NS
	TRN	16	18.31	
	Total	32		
After seven days	WaveOne Gold	16	16.50	1.00*NS
	TRN	16	16.50	
	Total	32		

## Discussion

Numerous methodologies have been used to assess postoperative pain. VAS was employed in this study due to its widespread use in research for assessing pain levels and established dependability [18-21]. This study only looks at mandibular molars. An isolated variable for preoperative pain was set to avoid bias, the variable being selecting teeth with the preoperative diagnosis of irreversible pulpitis to eliminate the possibility of intracanal drugs or other variables causing discomfort, and all of the teeth are treated in one visit [14]. The volume, concentration, and kind of irrigating solution used in all endodontic treatments, as well as root-filling operations, are all standardized [14]. As a result, both professionals and patients have thoroughly appreciated the importance of the VAS and its implementation [1]. The removal of dentin from root canal walls and the shaping of the canal before receiving filler material are the goals of root canal preparation. Because instruments cannot reach all dentinal walls, preparing root canals completely with an uneven architecture can be difficult [22].

The comprehensive study by Pak and White [3] found that the average level of discomfort experienced during root canal preparation peaked within the first 24 hours and then markedly dropped over the following days, particularly in the first two. This result is consistent with the current study. Another study having a consistent result with the current study is by Roshdy and Hassan [23]; their study (Quantitative evaluation of apically extruded debris using TRUShape, TruNatomy, and WaveOne Gold in curved canals) concluded that debris extrusion was less in WOG group when compared to TruNatomy and TRUShape (Dentsply Sirona, Tulsa, OK). They stated that future in vivo research comparing the prevalence to the degree of postoperative discomfort following mechanical preparation is necessary for further association, and the current study confirms their findings. The comparative study by Xavier et al. (Postoperative pain after use of the WaveOne Gold and XP-endo Shaper systems: a randomized clinical trial) [14] concluded that the reciprocating WaveOne Gold filing system shows less postoperative pain when compared to the rotatory XP-endo Shaper (FKG Dentaire, La Chaux-de-Fonds, Switzerland). Martins et al. [24] discovered that when compared to a rotating system, a reciprocating system causes reduced postoperative pain (p<0.05). In certain clinical research, contrary results have been found. Pasqualini et al.'s study (Postoperative quality of life following single-visit root canal treatment performed by rotary or reciprocating instrumentation: a randomized clinical trial) [17] compared WaveOne Gold to ProTaper and was conducted in 2015; another study was conducted in 2016 by Zand et al. [25]; in their study (Treatment of necrotic teeth using two engine-driven systems and patient's postoperative pain: a double-blind clinical trial), they compared RaCe to Reciproc. It showed that reciprocal motion has been linked to a higher rate of postoperative discomfort than continuous rotational motion, though this has yet to be demonstrated decisively; the reason for the contrary results might be that different filing systems are compared; also, the studies had different preoperative clinal diagnosis criteria for the selection of patients, whereas the recent studies show a consistent result with the current study.

Limitation of the study

The limitation of the study is pain being a subjective symptom and might result in bias depending on the pain threshold of the patient, which varies from individual to individual. According to the criteria set forth in this study, there was a statistically insignificant difference in postoperative discomfort between the preparation systems tested at 24 hours, 72 hours, and seven days.

## Conclusions

The study suggests that following the usage of the evaluated systems, short-term postoperative pain (lasting from 24 to 72 hours) can be anticipated, although no pain is anticipated after seven days. The average pain at the assessment times was categorized as mild in both groups, despite the fact that the WaveOne Gold system generated less postoperative discomfort than the TruNatomy filing system.
